# *Neobenedenia melleni*-Specific Antibodies Are Associated with Protection after Continuous Exposure in Mozambique Tilapia

**DOI:** 10.1155/2015/635387

**Published:** 2015-02-08

**Authors:** Jennifer M. Kishimori, Akihiro Takemura, Jo-Ann C. Leong

**Affiliations:** ^1^Hawaii Institute of Marine Biology, School of Ocean & Earth Science and Technology, University of Hawaii at Manoa, P.O. Box 1346, Kaneohe, HI 96744, USA; ^2^Sesoko Station, Tropical Biosphere Research Center, University of the Ryukyus, 3422 Sesoku, Motobu, Okinawa 905-0227, Japan

## Abstract

*Neobenedenia melleni* is a significant monogenean pathogen of fish in aquaculture facilities and public aquaria. Immunity after exposure to live *N. melleni* is well established, but the mechanisms of immunity remain unclear. In this study, tilapia (*Oreochromis mossambicus*) were continuously exposed to *N. melleni* over a four-month period and assessed for immunity as determined by a reduction in the number of parasites dislodged from the experimental animals during freshwater immersion. Specific mucosal and systemic antibody levels were by determined via enzyme-linked immunosorbent assay. At 45 days postexposure (DPE), fish displayed high parasite loads and baseline levels of mucosal antibodies. At 102 and 120 DPE parasite loads were significantly decreased, and antibody levels were significantly increased for mucus and plasma samples. The correlation between immunity (reduction in parasite load) and an increased humoral antibody response suggests a key role of antibody in the immune response. This is the first report of immunity against *N. melleni* that is associated with specific mucosal or systemic antibodies.

## 1. Introduction

**Figure 1:**
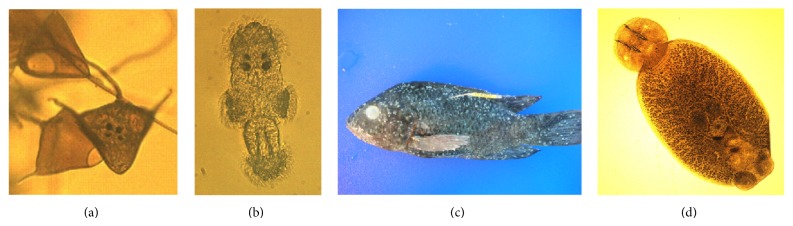
Life cycle of the Hawaiian* N. melleni*. (a) Recently hatched eggs and single egg with eyespots. (b) Oncomiracidia. (c) Infected tilapia. (d) Adult parasite.

*Neobenedenia melleni* (synonymous* N. girellae* Whittington and Horton [1]) is a monogenean ectoparasite that affects over 100 species of marine teleost families in aquaculture facilities and public aquaria (Whittington and Horton [1]). In Hawaii,* N. melleni* has been reported in sea cage-cultured tilapia (*Oreochromis mossambicus*) in Kaneohe Bay and was the source of an outbreak in sea cage-cultured amberjack (*Seriola rivoliana*) off the west coast of Oahu in 2004 (Kaneko II et al. [2] and Lewis and Kishimori [3]). The direct lifecycle of the Hawaiian* N. melleni* spans 12–16 days and can enable rapid amplification in culture facilities (Figure 1).

Acquired protection (immunity) against* N. melleni* following exposure has been well-documented but the basis for this immunity remains unclear (Nigrelli [4], Bondad-Reantaso et al. [5], Robinson et al. [6], and Ohno et al. [7]). There is evidence that the systemic humoral response may not be an important component of immunity against* N. melleni* (Bondad-Reantaso et al. [5], Hatanaka et al. [8], and Robinson et al. [6]). However, mucus from* N. melleni*-exposed fish has been shown to have* in vitro* antiparasitic effects (Nigrelli [4] and Robinson et al. [6]). Investigators have reported the induction of specific mucus antibodies in several teleost systems (Zhao et al. [9], Maki and Dickerson [10], and Vervarcke et al. [11]). Pathogen-specific mucus antibody associated with protection in fish has been shown for metazoans (Rogers-Lowery et al. [12]), protozoans (Luo et al. [13]), and bacteria (Esteve-Gassent et al. [14]). The reported* N. melleni*-killing effects of mucus and evidence of specific mucus antibody in other systems suggest that mucus from* N. melleni*-exposed fish may contain key protective factors, including specific antibody. The purpose of this study was to document the mucosal immune response associated with acquired protection (immunity) in the tilapia during continuous exposure to* N. melleni*.

## 2. Methods

### 2.1. General

Tilapia (*O. mossambicus*) was monitored for antibody responses during the development of immunity against* N. melleni* over a four-month period. The seawater used in all aspects of this experiment was treated via a sand filter, canister filters, and an ultraviolet system. All experiments were conducted in accordance with the principles and procedures approved by the Institutional Animal Care and Use Committee, University of Hawaii.

### 2.2. Parasite Propagation

Fomites (nylon nets) contaminated with* N. melleni* eggs from a commercial aquaculture facility were used to initially propagate* N. melleni* on tilapia to obtain a continuous source of parasites.

### 2.3. Fish Husbandry and Infection

Seventeen individually tagged, 1-2-year-old fish (12.1–16.5 ± 1.21 cm and 30.0–85.1 ± 14.6 g), raised in fresh water, and naïve to* N. melleni* were acclimated to seawater over a 5–7-day period and maintained in an outdoor 400-gallon fiberglass tank under flow-through conditions at natural photoperiod until exposure. Fish were fed once daily to satiation (Silver Cup Trout Chow, Nelson and Sons; Murray, UT). Fish were transferred to an indoor parasite challenge room and housed in a 30-gallon glass aquarium with a box filter (Marineland Penguin 200; Cincinnati, OH). Water changes (50–75%) were performed weekly or more often as needed and monitored as needed for temperature (25-26°C), pH (7.4–8.0), and NH_3_/NH_4_ (0–1.5 ppm) (Aquatic Pharmaceuticals Incorporated; Chalfont, PA). Fish were acclimated for 24 hours and cohabitated with an infected fish for two weeks, after which the infected fish was removed. Patency of infection was confirmed by observing viable* N. melleni* eggs on a 2 × 2 cm square of netting deployed in the tank weekly. The infection on the 17 fish was allowed to progress until evidence of an intense infection was apparent (lethargy, flashing, mucus hypersecretion, and corneal opacity), which occurred at 45 days postexposure (DPE).

### 2.4. Parasite Quantification

Fish were treated with a 10-minute fresh water dip (FWD) and sampled for parasite loads at 45, 102, and 120 DPE. Fish were returned to the same seawater tank after each treatment. Parasites dislodged during the FWD were filtered through a 25 *μ*m mesh (Aquatic Ecosystems; Apopka, FL), transferred into a 50 mL tube, and counted manually under a SZXY Olympus Stereomicroscope System (Olympus America; Melville, NY).

### 2.5. Sample Collection

Mucus and plasma samples were collected after each FWD. Mucus was collected by placing individual fish in a plastic bag with gentle agitation for 1 minute. After removing the fish, the mucus was eluted into 1.5 mL polypropylene tubes and centrifuged at 10,000 ×g for 10 minutes. The supernatant was distributed into 0.3 mL aliquots and stored at −25°C until use. Blood was collected from the caudal venous sinus via heparinized needle and syringe, transferred to 1.5 mL polypropylene tubes, allowed to clot overnight at 4°C, and centrifuged at 1500 ×g for 10 minutes. The supernatant plasma was distributed in 0.1 mL aliquots and stored at −25°C until use.

### 2.6. ELISA Assay

Specific antibodies were measured via enzyme-linked immunosorbent assay (ELISA) modified from published protocols (Bondad-Reantaso et al. [5], Hatanaka et al. [8], and Dominguez et al. [15]). Preliminary experiments were performed to determine optimal dilution factors of the antibodies, mucus, and plasma samples used in the ELISA. Sonicated parasite antigen for ELISA was generated from parasites collected from infected fish via a 10-minute FWD. Individual parasites were transferred to a 15 mL polypropylene tube with a transfer pipette, rinsed five times in PBS, sonicated on ice with a W-385 Sonicator (Heat Systems Ultrasonics; Farmingdale, NY) until an opaque suspension was achieved, and centrifuged at 15,000 ×g for 20 minutes at 4°C. The supernatant containing soluble antigen was separated and analyzed for total protein levels via the BCA (bicinchoninic acid) protein assay (Pierce; Rockford, IL). Antigen was distributed into aliquots and stored at −25°C until use. EIA plates, 96-well (Costar; Corning, NY), were coated with 50 *μ*g/mL (50 *μ*L/well) of parasite protein at 37°C for 1 hour, blocked with 200 *μ*L of 1% BSA in 0.05% Tween/PBS (PBS-T) for 2 hours at 25°C, and incubated with 50 *μ*L of diluted sample (plasma or mucus) overnight, followed by rabbit antisera against tilapia (*Oreochromis niloticus*) IgM (Dominguez et al. [15]) diluted 1 : 2500 in PBS-T for 1 hour at 25°C and donkey anti-rabbit IgG (Jackson Immunoresearch Laboratories; Westgrove, PA) diluted 1 : 1000 in PBS-T for 1 hour at 25°C. Fifty *μ*L of ABTS substrate (KPL; Gaithersburg, MD) was added and plates were read at 405 nm. Included on each plate were positive controls (hyperimmune plasma or mucus from a chronically infected fish) and negative controls (plasma or mucus from a naïve fish). PBS was used as the blank. Mucus was diluted 1 : 3 and plasma was diluted 1 : 2000 in PBS. Between each incubation step, plates were washed twice with PBS-T, followed by PBS, for a total of 3 washes.

### 2.7. Statistics

Group comparisons were performed using a one-way analysis of variance (ANOVA) and Student's *t*-test. Significant levels were set at *P* < 0.05. Group data are expressed as means ± S.D. Correlation of infection levels and mucus antibody in individual fish was performed using Kendall's *τ*. All calculations were performed using the statistical software JMP-7.0.2 (SAS Institute, 2007; Cary, NC).

## 3. Results

### 3.1. Continuous Exposure with Serial Treatment Induces Immunity

**Figure 2:**
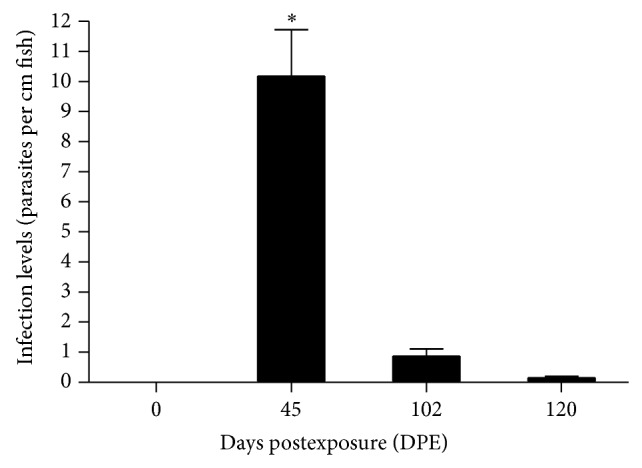
Effects of continuous exposure on infection levels as determined by number of parasites per fish length (cm) in tilapia at 45, 102, and 120 days postexposure (DPE) and uninfected fish (0 DPE). Vertical bars indicate mean ± SEM. ^*^Significantly different at *P* < 0.05, by one-way ANOVA and Student's *t*-test.

Fish displayed decreased parasite loads at 102 and 120 DPE when compared to 45 DPE (Figure 2). Mean infection levels at 45 DPE were 10.18 ± 6.37 parasites/cm fish or 146.47 ± 99.44 parasites/fish. At 102 DPE, these levels dropped significantly to 0.87 ± 0.98 parasites/cm fish or 12.19 ± 13.92 parasites/fish (*P* < 0.05). By 120 DPE, fish displayed marked immunity with 0.16 ± 0.15 parasites/cm fish or 2.19 ± 1.97 parasites/fish (*P* < 0.05).

### 3.2. Continuous Exposure Induces Parasite-Specific Mucosal and Systemic Antibodies

**Figure 3:**
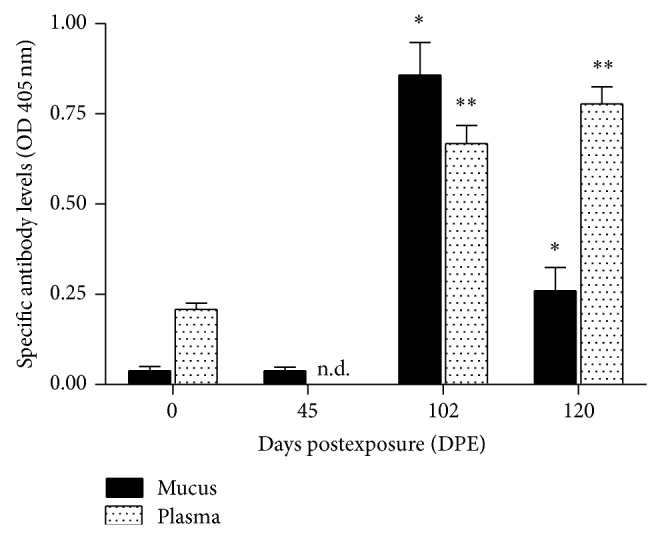
Effects of continuous exposure on specific antibody levels in tilapia mucus and plasma at 45, 102, and 120 days postexposure (DPE) and unexposed fish (0 DPE) as determined by ELISA and represented by optical density (405 nm). Mucus and plasma samples were diluted 1 : 3 and 1 : 2000 in PBS, respectively. Vertical bars indicate mean ± SEM. ^*^Significantly different from antibody levels in mucus of 0 DPE and 45 DPE groups at *P* < 0.05; ^**^significantly different from antibody levels in plasma of 0 DPE groups at *P* < 0.05 (one-way ANOVA and Student's *t* test). Blood samples were not collected at 45 DPE.

Specific mucosal and systemic antibodies were induced over the four-month period (Figure 3). Mucus antibody levels were at baseline levels in uninfected fish (0 DPE, OD 0.0383 ± 0.05) and, at 45 DPE (OD 0.0382 ± 0.04), increased significantly at 102 DPE (OD 0.8578 ± 0.36, *P* < 0.05) and then decreased to moderate levels but were still significantly above baseline at 120 DPE (OD 0.2600 ± 0.26, *P* < 0.05). Plasma antibody levels were increased at 102 DPE (0.6681 ± 0.20) and 120 DPE (0.7780 ± 0.19), both significantly higher than antibody levels of uninfected fish (0.2079 ± 0.08, *P* < 0.05). Blood samples were not taken for fish at 45 DPE.

### 3.3. Elevated Mucosal Antibodies Correlate with Decreased Parasite Loads

**Figure 4:**
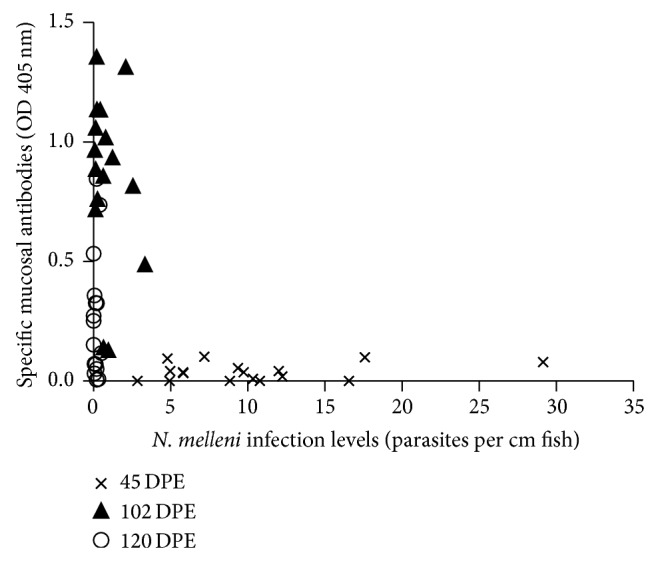
Relationship of specific mucosal antibodies to infection levels in continuously exposed tilapia as determined by nonparametric multivariate analysis. Antibody levels of mucus samples diluted 1 : 3 in PBS (optical density, 405 nm) and corresponding infection levels (parasites/cm fish) for individuals at each time point are indicated by × = 45 DPE, ▲ = 102 DPE, and ○ = 120 DPE. Mucosal antibodies and infection levels showed significant negative correlation (Kendall's *τ* = −0.3006, *r* = 0.4289, *P* < 0.01).

Elevated specific antibodies in mucus showed significant inverse correlation with infection levels in individual fish (Kendall's *τ* = −0.3006, *P* < 0.01, Figure 4). At 45 DPE, individuals displayed low levels of mucosal antibodies and high numbers of parasites; in contrast, at 102 DPE these fish exhibited high mucosal antibody levels with low parasite numbers and at 120 DPE fish displayed moderately elevated mucosal antibody levels corresponding to very low parasite loads.

## 4. Discussion

In the present study, tilapia continuously exposed to* N. melleni* displayed marked immunity and increased anti-parasite mucosal and systemic antibodies. Such marked immunity (e.g., a decrease in infection levels from a mean of 145 to 2 parasites per fish) against* N. melleni* has been documented only after multiple treatment protocols (Nigrelli [4] and Robinson et al. [6]).* N. melleni* infections rapidly amplify in closed systems and can reach pathogenic levels in susceptible species within one or two lifecycles (15–30 days) (Hirazawa et al. [16] and Jahn and Kuhn [17]). Yet, in the present study,* N. melleni* levels were extremely low after only two treatments (102 DPE). Further, mucosal antibodies and immunity appeared to be inversely related: mucosal antibodies peaked at about three months after exposure (102 DPE) with a concomitant drop in parasite loads. Significant immunity was achieved by four months after exposure with moderate mucosal antibody levels. We have observed decreased parasite survival times when incubated with hyperimmune mucus compared to naïve mucus (unpublished data). Nigrelli [4] and Robinson et al. [6] reported similar parasite-killing effects of mucus from fish recovered from* N. melleni* infection. Immunoglobulin-like proteins and parasite-killing effects have been shown in the mucus of soles (*Pleuronectes vetulus*) exposed to another ectoparasitic monogenean,* Gyrodactylus stellatus* (Moore et al. [18]). In the present study, the correlation of high specific mucosal antibody levels with significantly decreased* N. melleni* parasite loads suggests a key role for these antibodies.

Unexpectedly, continuous exposure with* N. melleni* also induced elevated specific systemic antibodies, which were observed at 3 and 4 months after exposure. It is unclear when systemic antibodies appear because plasma data from 45 DPE is not available. However, the spike in anti-*N. melleni* antibody levels in the mucus during sustained plasma antibody levels parallel findings in previous studies. Surface exposure of sunfish (*Lepomis macrochirus*) to the larvae of the parasitic bivalve (*Utterbackia imbelli*) induced systemic antibodies at 10 days post-infection that were observed every 10 days through day 80 while mucosal antibody production was only observed as a single spike at day 60 (Rogers-Lowery et al. [12]). Similarly, surface exposure of catfish (*Ictalurus punctatus*) to the protozoan* Ichthyophthirius multifiliis* induced serum antibodies at 5 weeks and a transient mucosal antibody response at 7 weeks after infection (Maki and Dickerson [10]). To our knowledge, the present study is the first report of specific systemic antibodies associated with protection against* N. melleni*.

The continual increase of plasma antibody levels in conjunction with decreased antibody levels in mucus and lower parasite loads also suggests a switch in the source of antibody from local production (epidermis) to systemic production. Phagocytosis of fluorescent microspheres by epidermal cells during immersion has been documented in fish, supporting the presence of antigen-presenting cells (APCs) in the epidermis (Kiryu et al. [19]). Epidermal antigen-specific B cells and plasma cells are upregulated after surface exposure with* I. multifiliis*, consistent with local antibody production in response to an ectoparasitic infection (Zhao et al. [9]). Further, surface exposure with* I. multifiliis* induces appearance of specific antibodies in serum and mucus, supporting the premise that antigen-specific B cells are home to both the anterior kidney and epidermis following infection (Maki and Dickerson [10] and Dickerson [20]). We suggest that, during continuous* N. melleni* exposure, epidermal APCs process antigens locally, inducing mucosal antibody production, and that APCs which migrate to the anterior kidney and spleen induce systemic antibody which may have increased avidity when compared to locally produced antibody. Lower levels of antibody at the host-parasite interface are thus sufficient to affect pathogen killing. This may also explain the inverse levels of mucus and plasma antibody levels at 3 and 4 months after exposure while immunity continued to improve.

Several studies have shown evidence against the role of antibodies in* N. melleni* immunity. Flounder (*Paralichthys olivaceus*) previously infected with* N. girellae* (*melleni*) exhibited immunity after challenge but displayed baseline levels of serum antibodies; intraperitoneal (IP) vaccination with sonicated parasites induced specific serum antibodies but was not protective (Bondad-Reantaso et al. [5]). IP vaccination of flounder with* N. girellae* (*melleni*) cilia induced specific antibodies in mucus and serum capable of agglutinizing/immobilizing oncomiracidia, yet these fish were not protected against challenge (Hatanaka et al. [8]). Continuously infected and treated hybrid tilapia displayed immunity at four months after exposure but was negative for specific serum antibodies on immunodiffusion (Robinson et al. [6]). In the present study, fish were not treated until overt signs of ectoparasitism were observed, suggesting that antigen exposure route, time, and intensity may be critical for antibody induction. Unlike locally invasive pathogens such as* I. multifiliis* and* U. imbelli*,* N. melleni* appears to graze on the epidermis, likely decreasing its accessibility to the systemic immune system (Sato et al. [21]). This may be one explanation why previous studies on* N. melleni* have not identified specific antibodies after surface exposure; the exposure may not have been intense or long enough to induce mucus or serum antibody production.

There are a number of limitations with the present study. The exact initial exposure dose for each fish is unknown as a single infected fish was cohabitated with the experimental fish for one life cycle of the parasite. However, this design best modeled the scenarios within aquaria where an infected and immune fish, displaying no clinical signs of infection, is introduced to a naïve population in a closed system, and a parasite bloom is then observed weeks or even months later. This design also achieved the goal of continual, intense exposure with multiple sampling of the same individual fish, which is not possible if cohorts are removed for sampling and then euthanized. In addition, large sampling intervals were implemented in this study, initially based on the first time point, which was dependent on the infection intensity; however, only mucus samples were collected at that time point. Shorter, regular sampling intervals for parasite loads, mucus, and blood would enable a more complete assessment on the mucosal and systemic antibody response to this parasite. Nonetheless, this study revealed individual fish displaying low levels of parasites in the presence of high levels of mucosal and systemic antibodies after intense infection levels and low mucus antibody levels.

Without functional experiments, the role of the anti-*N. melleni* antibodies in immunity is still unclear; the protective effects of mucus* in vitro* and* in vivo* must be proven. The antibody response may not be the most important aspect of protection and the role of innate and cell-mediated mechanisms cannot be ruled out. Recently, several studies have focused on the host-parasite interface, reporting that* N. melleni* induces epidermal thinning and mucus cell hyperplasia in amberjack (*Seriola dumerili*) (Hirayama et al. [22]). Indeed, host mechanisms at the parasite interface are likely critical to the induction of a protective response against true ectoparasites like* N. melleni*. If properly stimulated, the mucosal immune system may orchestrate significant protection via cytokine release, leukocyte induction, cellular homing signals, and antibody production as described for mammalian mucosal immunity (Buchmann [23]).

Vaccine development classically relies on optimal antigen identification, primarily via induction of specific immunoglobulins. If, indeed, fish consistently develop protective mucosal and systemic antibodies following prolonged infection against* N. melleni*, it may be possible to identify key antigens for vaccination efforts or immunological surveys to enable risk assessments. In the last few years, there has been increased interest in the host-parasite interface for* N. melleni*; further,* N. melleni* adhesive material proteins have recently been identified that may be involved in the host mucosal immune response and could lay the basis for antigen discovery (Maffioli et al. [24]). Specific mucosal immune responses may be integral to this discovery, to include production of protective antibody. To our knowledge, this is the first report of specific mucosal and systemic antibodies against* N. melleni* that is associated with a protective status in the host.
